# Mesenteric Plexiform Neurofibroma as a Cause of Weight Loss and Chronic Diarrhea in a Patient with *YPEL3* Variant

**DOI:** 10.1097/PG9.0000000000000098

**Published:** 2021-07-12

**Authors:** Irina Gorbounova, Arthur Lenahan, Tara Lynn Wenger, Erin Rudzinski, Elizabeth Ren-Yee Tang, Caitlin A. Smith, Danielle Wendel, Simon Horslen, Lusine Ambartsumyan

**Affiliations:** From the * Division of Gastroenterology and Hepatology, Seattle Children’s Hospital, University of Washington School of Medicine, Seattle, WA; † Seattle Children’s Hospital, University of Washington School of Medicine, Seattle, WA; ‡ Division of Genetic Medicine, Seattle Children’s Hospital, University of Washington School of Medicine, Seattle, WA; § Department of Laboratories, Seattle Children’s Hospital, University of Washington School of Medicine, Seattle, WA; ∥ Department of Radiology, Seattle Children’s Hospital, University of Washington School of Medicine, Seattle, WA; ¶ Department of Surgery, Seattle Children’s Hospital, University of Washington School of Medicine, Seattle, WA.

**Keywords:** malnutrition, genetics, tumor

## Abstract

Mesenteric plexiform neurofibroma is a subtype of plexiform neurofibroma that involves the mesentery and causes a variety of gastrointestinal complaints. Plexiform neurofibroma is classically found in patients with neurofibromatosis type 1, although genetic contributions to plexiform neurofibroma pathogenesis are heterogeneous. We report the first case of mesenteric plexiform neurofibroma in a patient with a *YPEL3* pathogenic variant. This patient presented with growth failure, generalized abdominal pain and chronic diarrhea. She was confirmed to have mesenteric plexiform neurofibroma on histopathology and targeted sequencing on affected tissue confirmed that there were no neurofibromatosis type 1 variants present. Given that this patient’s mesenteric plexiform neurofibroma is associated with *YPEL3* dysfunction, she is unlikely to benefit from MEK inhibitors, which are the newly approved treatment for inoperable plexiform neurofibroma in patients with neurofibromatosis type 1.

## INTRODUCTION

Plexiform neurofibroma (PN) is a benign tumor that develops along nerve fascicles with potential for malignant transformation, classically described in patients with neurofibromatosis type 1 (NF1) (1). Mesenteric plexiform neurofibromas (MesPN) can be asymptomatic or present with variable gastrointestinal complaints, including abdominal pain, weight loss, diarrhea, ulcers, intestinal obstruction, intussusception, and volvulus (1, 2). We report the first case of a patient with a *YPEL3* pathogenic variant with diarrhea and malnutrition associated with a solitary non-NF1-related MesPN. Informed consent was obtained before manuscript submission.

## CASE REPORT

A 16-year-old female with germline pathogenic variant in *YPEL3*, hypomyelination and hypertrophic peripheral nerves, motor neuropathy with hyporeflexia and hypotonia, remote history of lymphedema, neuromuscular scoliosis, hip dysplasia, restrictive pulmonary disease, and gastroesophageal reflux requiring fundoplication and gastrostomy feeding presented with faltering growth, abdominal pain, and chronic diarrhea. Whole exome sequencing revealed that her baseline clinical features were associated with a novel frameshift mutation of *YPEL3* resulting in loss of function (3). Severe postprandial diarrhea was associated with generalized abdominal pain and required admission for malnutrition after losing 6.7 kg in 6 months (weight z score –7.35). On presentation, she had 10–15 bowel movements daily. Trials of elemental and whole-food formulas had no symptomatic improvement. Loperamide slightly improved her diarrhea, although enteral intake provoked her abdominal pain.

**FIGURE 1.**
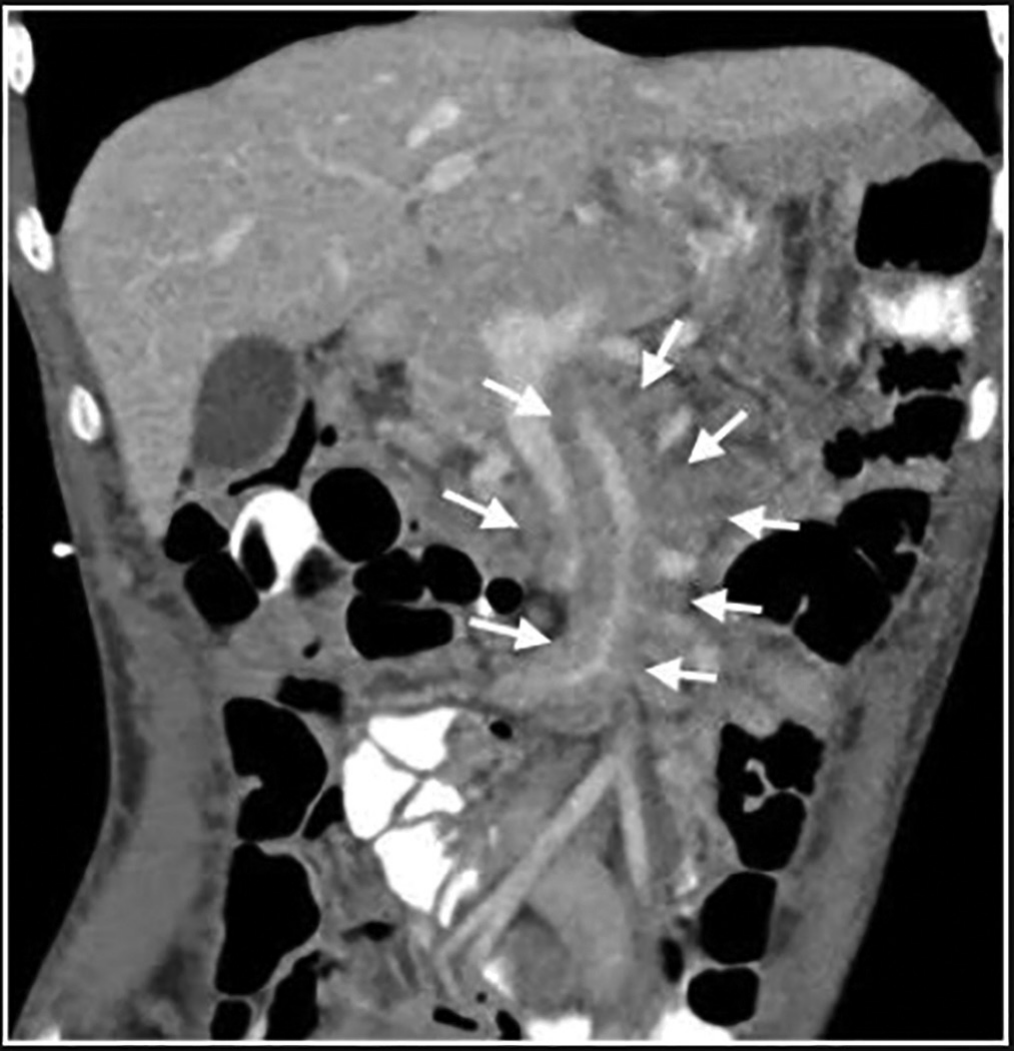
Coronal image from contrast-enhanced abdominal computed tomography shows abnormal diffuse soft tissue infiltration (arrows) throughout the mesentery, particularly around the mesenteric vessels.

She had a normal complete blood count (CBC), comprehensive metabolic panel, inflammatory markers, and thyroid studies. She had normal fecal elastase, stool alpha-1-antitrypsin, infectious stool studies, and serology for neuroendocrine tumors (somatostatin, vasoactive intestinal peptide, gastrin, and serotonin levels). Esophagogastroduodenoscopy was visually normal with mild chronic gastritis on histopathology. Colonoscopy was visually normal with patchy moderately active colitis on histopathology. Capsule endoscopy revealed normal intestinal mucosa. Findings were not consistent with inflammatory bowel disease (IBD) due to lack of chronic inflammation on histopathology, normal C-reactive protein, and CBC. She underwent a trial of steroid enemas with no symptomatic improvement. Gastric emptying study was normal. Abdominal computed tomography (CT) showed diffuse infiltrative soft tissue density around the superior mesenteric artery and inferior mesenteric artery (Fig. 1).

**FIGURE 2.**
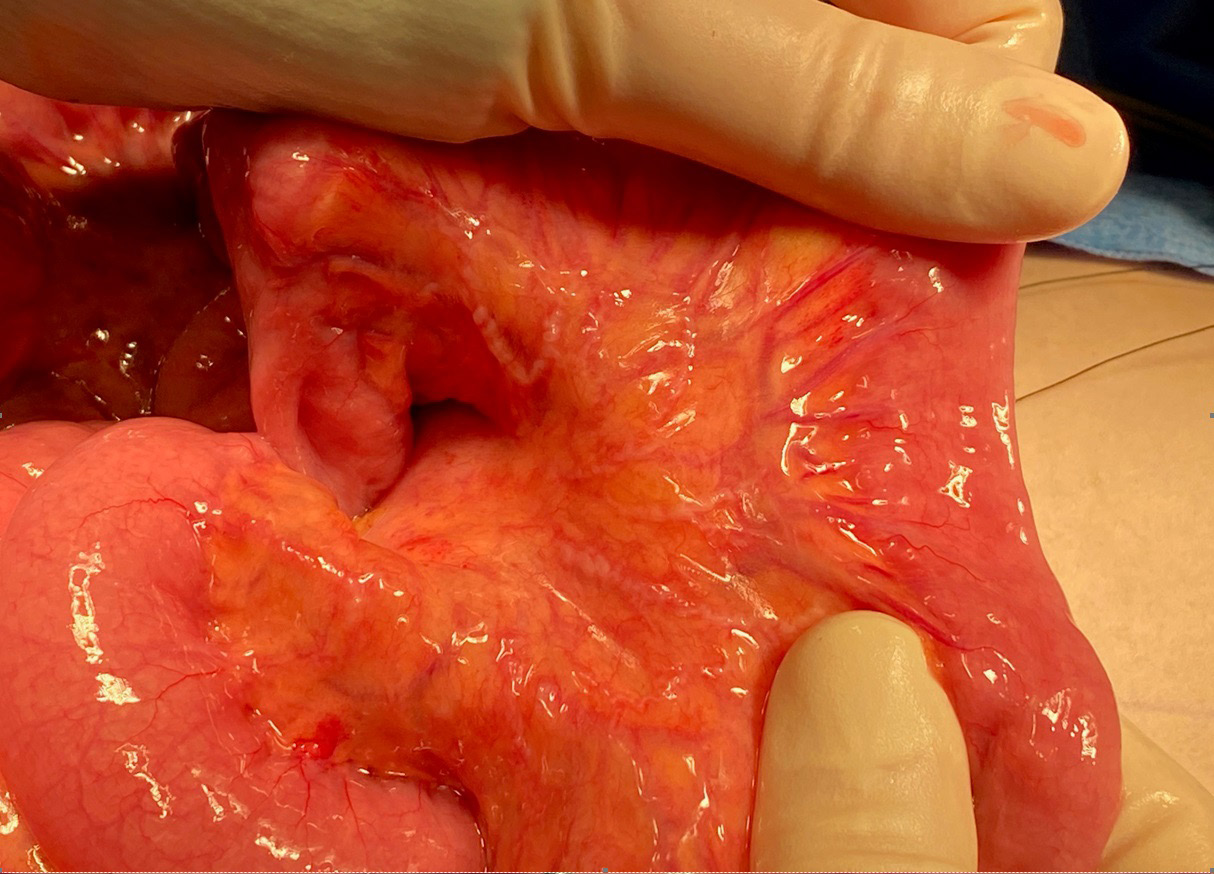
Thickened mesentery with nodular cord–like structures on gross examination.

**FIGURE 3.**
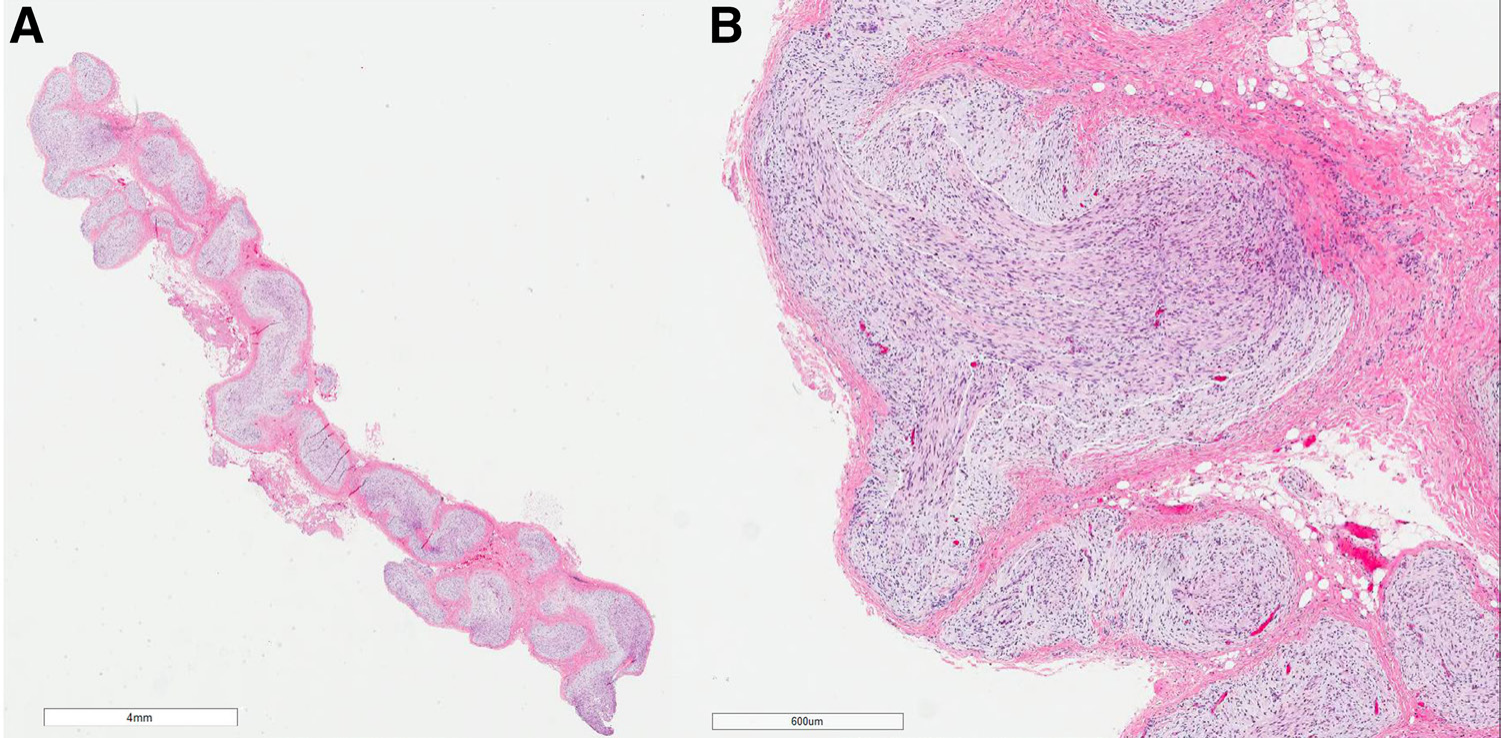
Histologic examination showed tortuous expansions of peripheral nerve ([A] hematoxylin and eosin stain) characterized by spindled cells in a myxoid background surrounded by perineurium ([B] hematoxylin and eosin stain).

A laparotomy was performed to obtain tissue based on the abdominal CT findings. The entire mesentery was thickened with nodular cord–like structures (Fig. 2). Complete surgical resection was not possible due to extensive mesenteric involvement. Tissue was obtained from the mass, confirming PN without malignancy (Fig. 3). As PNs are typically seen in NF1, she underwent a clinical evaluation for signs of NF1 and had no clinical features consistent with the complete or segmental NF1. Targeted sequencing on affected tissue confirmed that there were no *NF1* variants present. Our patient was started on parenteral nutrition, providing 90 kcal/kg/d with excellent weight gain. Diarrhea and pain resolved after minimizing oral intake.

## DISCUSSION

This case demonstrates MesPN in a patient with malnutrition and diarrhea associated with pathogenic loss-of-function variant in *YPEL3.* PN is a benign peripheral nerve tumor that develops along nerve fascicles with an unpredictable growth pattern. It can involve any peripheral nerve, typically consisting of multiple nerve fascicles, and occurs in various locations including subcutaneous tissue, craniofacial, and retroperitoneum (1). When PN involves the mesentery, it likely arises from Auerbach’s plexus or mesenteric autonomic nerves (1,2). MesPN consists of Schwann cells, mast cells, endothelial cells, perineural cells, and fibroblasts (4). PN can rarely transform into a malignant peripheral nerve sheath tumor, a soft-tissue sarcoma with poor prognosis (5).

PN had been thought to be pathognomonic for NF1. However, an estimated 10% of patients with PN were described without pathogenic variant in *NF1*, specifically in cases of craniofacial PN (6). This patient underwent clinical evaluation for signs of complete or segmental NF1 and also had sequencing of *NF1* in affected tissue, confirming that this was not the cause of her MesPN.

Treatment for MesPN is limited. Complete surgical resection is advised, although often challenging due to infiltration of mesentery (1,2). MEK inhibitors, which target RAS/MAPK pathway, have recently been approved for inoperable NF1-related PN (4). Our patient did not qualify for treatment with MEK inhibitors, as her MesPN is not due to known disruption of signaling through the RAS/MAPK pathway. Genetic contributions to PN pathogenesis are heterogeneous and poorly understood (5), although in our patient, it is likely attributable to her loss-of-function variant in *YPEL3.*

*YPEL3* is involved in differentiation of myelinating cells, including perineurial glia and Schwann cells (3). Canonical Wnt signaling cascades are an important tumor suppressor pathway shown to be altered in human malignancies and in PN (7). *YPEL3* silencing has been indirectly shown to disinhibit the Wnt pathway by stabilizing β-catenin, thus inducing epithelial-mesenchymal cell transition, leading to tumorigenesis (8). Additionally, mutations in *YPEL3* result in a lack of functional myelinating cells and improper intercellular binding during the compaction stage of myelination, leading to hypertrophied and hypomyelinated nerve fascicles (3). We suspect that in our patient, MesPN and the resulting gastrointestinal symptoms are associated with dysregulated myelinating cell growth and defective Wnt signaling due to *YPEL3* dysfunction (7). The mechanism for diarrhea and growth failure remains unknown (1,2). We speculate that abnormal enteric nervous system innervation and altered modulation by the autonomic nervous system result in abnormal gastrointestinal motility, absorption, and secretory function clinically presenting as diarrhea and malnutrition.

Patchy moderately active colitis on histopathology could be a result of MesPN or early IBD. Resolution of her abdominal pain and diarrhea with decreased oral intake, normal inflammatory markers, and CBC disfavors IBD. A repeat colonoscopy and fecal calprotectin would be warranted if she continues to have symptoms with oral introduction of feeds to assess for possible developing IBD.

Given that our patient’s MesPN is likely driven by Wnt pathway, she would be unlikely to benefit from treatment with a MEK inhibitor. She is currently asymptomatic and gaining weight on parenteral nutrition over the past year.
